# Ovarian Torsion in Normal Ovaries in Reproductive-Aged Women

**DOI:** 10.1155/crog/6630642

**Published:** 2025-09-02

**Authors:** Bushra Zaman Bandhon, Vinita Rajadurai, Cherynne Johansson

**Affiliations:** Obstetrics and Gynaecology Department, Liverpool Hospital, Sydney, New South Wales, Australia

**Keywords:** detorsion, Doppler ultrasound, laparoscopy, normal ovaries, ovarian torsion

## Abstract

Ovarian torsion is a gynecological emergency caused by the rotation of an ovary on its supporting ligaments, which can obstruct blood flow and lead to ovarian necrosis if untreated. Typically, torsion is associated with ovaries enlarged by cysts or masses, which increase the likelihood of rotation around the supporting ligaments. Although torsion can also occur in normal ovaries, especially in premenarchal girls with elongated infundibulopelvic ligaments, literature on this occurrence in reproductive-aged women is limited. This report presents two cases of ovarian torsion in normal ovaries, highlighting diagnostic and management challenges. The first case involves a 19-year-old who presented with acute right iliac fossa pain. Ultrasound showed a normal ovary with intact blood flow. However, due to ongoing pain, she underwent laparoscopy, which confirmed a 1.5-twist torsion at the utero-ovarian ligament. Right ovarian detorsion was performed successfully, and her postoperative recovery was uneventful, with follow-up ultrasound showing no abnormalities. The second case describes a 40-year-old woman with a prior hysterectomy, presenting with left iliac fossa pain. Ultrasound showed an enlarged, heterogeneous left ovary (5 × 3.2 × 4.2 cm) with poor blood flow and complex free pelvic fluid, raising suspicion for torsion. Emergency laparoscopy confirmed a 2-twist torsion on the infundibulopelvic ligament. Despite attempts at detorsion, the ovary remained nonviable, leading to left oophorectomy. These cases emphasize that ultrasound and Doppler findings may not reliably detect torsion in normal ovaries. Laparoscopy remains the definitive method for diagnosis and intervention, providing timely treatment that is essential to preserve ovarian function. Clinicians should maintain a high suspicion for torsion in reproductive-aged women with acute abdominal pain, even if imaging is inconclusive, to prevent severe complications. These cases highlight the need for heightened awareness of ovarian torsion in normal ovaries to optimize surgical outcomes and fertility preservation.

## 1. Introduction

Ovarian torsion is a medical emergency characterized by the complete or partial rotation of the ovary on its supporting ligaments. The underlying pathophysiology involves a reduction in venous return, stromal edema, internal hemorrhage, and infarction with the subsequent sequelae. This condition is one of the most common gynecological surgical emergencies and can affect females of all ages. Ovarian torsion typically occurs when an ovarian cyst or mass enlarges the ovary greater than 5 cm in diameter, causing the ovary to rotate around the infundibulopelvic and utero-ovarian ligaments [1]. These ovarian cysts or masses are usually benign. Although torsion can occur in normal ovaries, especially in premenarchal girls with elongated infundibulopelvic ligaments, there is limited literature on ovarian torsion in normal ovaries of reproductive-aged women [2]. Here, we present two cases of ovarian torsion in normal ovaries with no cysts or masses in reproductive-aged women.

## 2. Case Reports

### 2.1. Case 1

A 19-year-old female presented to the emergency department with a history of sudden onset sharp right iliac fossa pain. She denied any associated nausea, vomiting, or fevers. Her bowel and bladder functions were normal. She was not sexually active, had never been pregnant, and had no history of sexually transmitted infections or pelvic inflammatory disease. Her medical, surgical, and gynecological histories were unremarkable. Her menstrual periods were always regular, and she was due for her next period.

On examination, she was vitally stable. Her abdomen was soft and tender in the right iliac fossa, with no guarding or rigidity. Pelvic ultrasound showed a bulky right ovary measuring 58 × 34 × 50 mm (54 mL), with flow observed within the ovary. The left ovary was normal, but a large amount of free pelvic fluid was noted, suggesting intermittent torsion of the right ovary.

**Figure 1:**
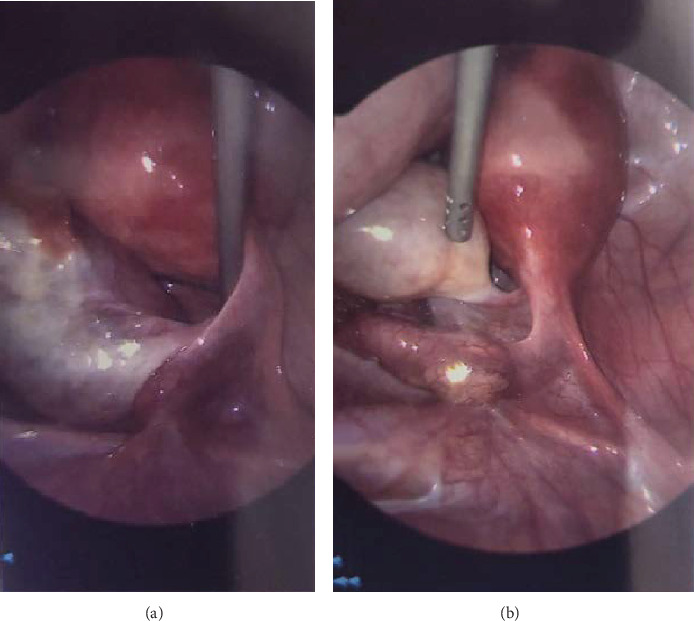
(a) Bulky right ovary with torsion at the utero-ovarian ligament. (b) Right ovarian detorsion.

The working diagnosis was intermittent torsion of the right ovary +/− cyst accident. She was admitted for overnight observation. The following morning, due to ongoing pain, she was taken to the theatre. Laparoscopy revealed a bulky right ovary with torsion at the utero-ovarian pedicle x1.5 (Figure 1a). Right ovarian detorsion was performed (Figure 1b). The left ovary and bilateral fallopian tubes appeared normal, and the utero-ovarian ligaments were not elongated.

Postoperatively, she recovered well and was discharged the following day with advice to follow up in the outpatient gynecology clinic. On her postoperative follow-up in the clinic, she was noted to be doing well and her postoperative repeat pelvic ultrasound was normal. The cytology of the peritoneal fluid did not find any malignant cells. She was discharged from the gynecology clinic. Written informed consent was taken from the patient to publish her case in the journal.

### 2.2. Case 2

A 40-year-old woman presented to the hospital with a history of sudden onset severe left iliac fossa pain, following a hysterectomy 2 years prior. On presentation, she was vitally stable with tenderness in the left iliac fossa and left flank but no signs of peritonitis.

Her blood tests were unremarkable. CT abdomen and pelvis revealed a peripherally enhancing multiloculated cystic right adnexal lesion with the largest loculated component measuring up to 36 × 32 mm and a small amount of pelvic fluid, suggesting a possible cyst accident or tubo-ovarian abscess (TOA). Pelvic ultrasound revealed an enlarged heterogeneous left ovary with associated complex free fluid in the pelvis, with poor flow on Doppler studies, raising concerns for a left ovarian torsion. The ovary measured 5 × 3.2 × 4.2 cm and possibly contained a hemorrhagic cyst.

The working diagnosis was ovarian cyst/torsion. Given her well-controlled pain and remaining stable, she was admitted for overnight observation, with a plan to fast from midnight and a possible diagnostic laparoscopy +/− detorsion/cystectomy/salpingo-oophorectomy if the pain worsened. Her pain worsened overnight, necessitating increased analgesia and leading to emergency surgery being performed.

**Figure 2:**
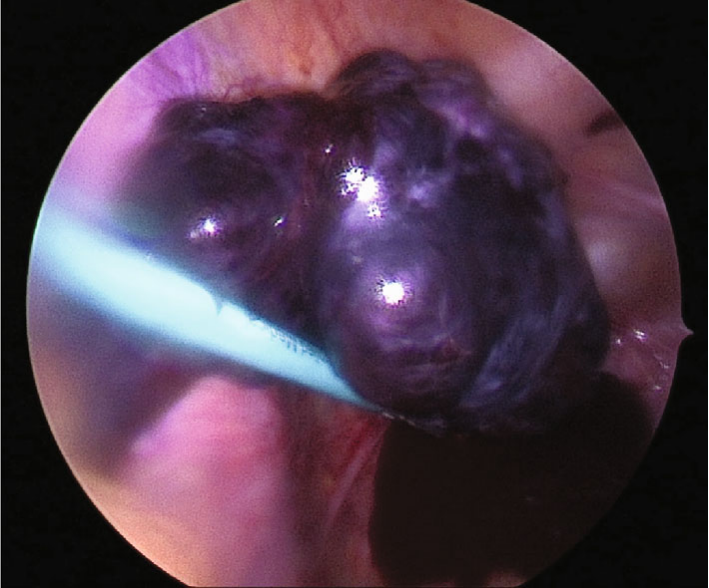
Edematous, congested, necrotic-looking left ovary with blood in the pelvis.

Intraoperatively, torsion of the left ovary on the infundibulopelvic ligament x2 was noted. The left ovary appeared enlarged, edematous, congested, and hemorrhagic (Figure 2). A period of observation for 5 min was done following detorsion of the ovary. However, as there were no signs of improvement in the ovary's appearance, indicating nonviability, a left oophorectomy was performed. The right ovary, bowel, and appendix appeared normal.

Postoperatively, she remained well and was discharged the following day with a plan for follow-up in the outpatient gynecology clinic. On her 6 weeks' postoperative follow-up visit, she was noted to have recovered well. Her histopathology result revealed features compatible with torsion. However, she mentioned experiencing new-onset intermittent right iliac fossa pain, which prompted her general practitioner to organize a pelvic ultrasound. The pelvic ultrasound revealed a 5 × 3.3 × 3.7 cm simple cyst in the right ovary, with no adnexal mass or free fluid. She has been booked for a laparoscopic cystectomy considering the size of the cyst and the increased risk of torsion. Written informed consent was taken from the patient to publish her case in the journal.

## 3. Discussion

Ovarian torsion can affect females of all ages, including fetuses and neonates, especially when an ovarian mass is present. However, most of the ovarian torsion occurs in the reproductive age and is less common in the premenarchal and postmenopausal group (17.2% of cases) [3]. In adults, common predisposing factors include ovarian physiological cysts, ovulation induction, or benign neoplasms. In fact, over 80% of torsion cases involve ovarian masses of 5 cm or larger [4]. The presence of an ovarian mass increases the likelihood of the ovary rotating on the axis of the infundibulopelvic and utero-ovarian ligaments, leading to torsion, although it can occur in ovaries of any size, including normal ovaries without a cyst or mass.

A retrospective case series conducted by Tabbara et al. [5] at a tertiary care emergency department reported that 75% of ovarian torsion cases occurred in patients with an ovarian cyst or mass. This finding reinforces the well-established association between adnexal pathology and torsion, highlighting the rarity of ovarian torsion in morphologically normal ovaries, particularly in reproductive-aged women [5].

Similarly, Karaman et al. [6] studied postmenarchal adolescent girls and found that only 27% of torsion cases involved normal ovaries. They further emphasized the diagnostic challenges in this subgroup, noting that the absence of structural ovarian pathology often leads to delays in diagnosis, potentially compromising ovarian viability [6].

These studies collectively underscore the diagnostic dilemma and clinical uncertainty in cases of ovarian torsion without identifiable cysts or masses, particularly in young or reproductive-aged patients.

Our two case reports illustrate instances of ovarian torsion occurring in normal ovaries without the presence of a cyst or mass. Fixed ovaries, such as those due to endometrioma, TOA, or malignancy, are less likely to undergo torsion. Ovarian torsions are more likely to occur with benign masses than in malignancies. The incidence of ovarian torsion with ovarian malignancy was < 2% in reported case series [7].

Recurrence of ovarian torsion is uncommon. However, those who have torsion in the absence of an ovarian mass may have a higher risk of recurrence than those with an ovarian mass [8].

The diagnosis of ovarian torsion is challenging due to the variable clinical presentations. Patients often report sudden onset pain, nausea, vomiting, and low-grade fever. Ultrasound is a key diagnostic tool, although its sensitivity ranges from 46 to 75% [9]. A torted ovary may appear rounded, enlarged, and heterogeneous due to edema, engorgement, or hemorrhage [10]. The presence of Doppler flow does not exclude torsion as flow can be normal, decreased, or absent, due to incomplete occlusion or collateral blood supply [11]. The “whirlpool sign” on ultrasound, indicative of twisted ovarian vessels, is highly suggestive of torsion [12]. Magnetic resonance imaging (MRI) is expensive but can be helpful if ultrasound findings are equivocal [13].

In our two presented cases, on ultrasound, the torted ovaries were enlarged, bulky, and heterogeneous. Both cases reported free pelvic fluid. However, Doppler flow was normal in one case and poor in the other.

Laparoscopy is the preferred surgical approach due to its advantages of shorter hospital stay and reduced postoperative pain. However, laparotomy may be required in cases where there is suspicion of malignancy or when surgical skills necessitate. Traditionally, adnexectomy was the standard treatment for necrotic ovaries due to concerns about potential pulmonary embolism from untwisting a thrombosed ovarian vein. More recently, conservative management, including detorsion and cystectomy or cyst aspiration, has been advocated, even for necrotic-appearing ovaries. This approach has been shown to be safe and effective, preserving ovarian function and fertility. Studies support conservative management, showing no severe complications such as embolism or infection even after detorsion of necrotic-looking ovaries [14].

For premenopausal patients, if the ovary is viable, the next step is to assess for malignancy. If there is no concern for malignancy and a benign mass or large cyst is present, cystectomy or cyst drainage may be performed; otherwise, detorsion alone is sufficient [15]. If malignancy is suspected or the ovary is not viable, salpingo-oophorectomy is recommended. In postmenopausal patients, salpingo-oophorectomy is the preferred approach regardless of ovarian viability or the presence of cysts. This structured approach is aimed at preserving ovarian function in premenopausal women while ensuring appropriate surgical intervention when necessary.

## 4. Conclusion

These two case reports highlight the critical nature and diagnostic challenges of ovarian torsion, particularly in cases where torsion occurs in normal ovaries without the presence of a cyst or mass. Both patients, a 19-year-old and a 40-year-old, presented with acute onset abdominal pain and had varying Doppler flow findings. Despite these differences, both required prompt surgical intervention to address the torsion.

These cases underscore the importance of rapid diagnosis and intervention in ovarian torsion to optimize outcomes. They also highlight the need for awareness among clinicians regarding the potential for ovarian torsion in normal ovaries, ensuring prompt surgical consultation and intervention to preserve ovarian function and prevent severe complications.

## Data Availability

The data that support the findings of this study are available on request from the corresponding author. The data are not publicly available due to privacy or ethical restrictions.
